# MR spectroscopy-based brain metabolite profiling in propionic acidaemia: metabolic changes in the basal ganglia during acute decompensation and effect of liver transplantation

**DOI:** 10.1186/1750-1172-6-19

**Published:** 2011-05-09

**Authors:** James E Davison, Nigel P Davies, Martin Wilson, Yu Sun, Anupam Chakrapani, Patrick J McKiernan, John H Walter, P Gissen, Andrew C Peet

**Affiliations:** 1 Birmingham Children's Hospital NHS Foundation Trust, Birmingham UK; 2 University of Birmingham, Edgbaston UK; 3 Willink Biochemical Genetics Unit, Manchester UK

## Abstract

**Background:**

Propionic acidaemia (PA) results from deficiency of Propionyl CoA carboxylase, the commonest form presenting in the neonatal period. Despite best current management, PA is associated with severe neurological sequelae, in particular movement disorders resulting from basal ganglia infarction, although the pathogenesis remains poorly understood. The role of liver transplantation remains controversial but may confer some neuro-protection. The present study utilises quantitative magnetic resonance spectroscopy (MRS) to investigate brain metabolite alterations in propionic acidaemia during metabolic stability and acute encephalopathic episodes.

**Methods:**

Quantitative MRS was used to evaluate brain metabolites in eight children with neonatal onset propionic acidaemia, with six elective studies acquired during metabolic stability and five studies during acute encephalopathic episodes. MRS studies were acquired concurrently with clinically indicated MR imaging studies at 1.5 Tesla. LCModel software was used to provide metabolite quantification. Comparison was made with a dataset of MRS metabolite concentrations from a cohort of children with normal appearing MR imaging.

**Results:**

MRI findings confirm the vulnerability of basal ganglia to infarction during acute encephalopathy. We identified statistically significant decreases in basal ganglia glutamate+glutamine and N-Acetylaspartate, and increase in lactate, during encephalopathic episodes. In white matter lactate was significantly elevated but other metabolites not significantly altered. Metabolite data from two children who had received liver transplantation were not significantly different from the comparator group.

**Conclusions:**

The metabolite alterations seen in propionic acidaemia in the basal ganglia during acute encephalopathy reflect loss of viable neurons, and a switch to anaerobic respiration. The decrease in glutamine + glutamate supports the hypothesis that they are consumed to replenish a compromised Krebs cycle and that this is a marker of compromised aerobic respiration within brain tissue. Thus there is a need for improved brain protective strategies during acute metabolic decompensations. MRS provides a non-invasive tool for which could be employed to evaluate novel treatments aimed at restoring basal ganglia homeostasis. The results from the liver transplantation sub-group supports the hypothesis that liver transplantation provides systemic metabolic stability by providing a hepatic pool of functional propionyl CoA carboxylase, thus preventing further acute decompensations which are associated with the risk of brain infarction.

## Background

Propionic acidaemia (PA, OMIM #606054) results from deficiency of the biotin-dependent mitochondrial enzyme propionyl CoA carboxylase. The commonest and most severe form of PA presents in the neonatal period with acute metabolic acidosis, hyperammonaemia and progressive encephalopathy. Despite optimal management, later neurologic sequelae are a major cause of morbidity, and include neurodevelopmental delay, episodic acute encephalopathy, and movement disorders due to a propensity to basal ganglia infarction [1]. The mainstays of current treatment are dietary protein restriction, carnitine supplementation, and aggressive management of acute metabolic derangements. Some centres utilise alternative pathway drugs including sodium benzoate to minimise the consequences of hyperammonaemia. Orthotopic liver transplantation may prevent neurocognitive decline [2] but its role remains controversial.

The pathogenesis of the neurological insult remains poorly understood. Neurological damage may occur secondary to hyperammonaemia; alternatively inhibition of the mitochondrial Krebs cycle and electron transfer chain by accumulating metabolites may lead to a "bioenergetic stroke" [3]. Based on alterations in plasma amino acids associated with hyperammonaemia it has been hypothesised that the use of dietary supplements including glutamine that can replenish the Krebs cycle (anaplerotic therapy) may support aerobic cellular energy metabolism and hence improve clinical outcome [4]. Conflicting changes in cerebrospinal fluid glutamine concentrations have been reported in PA [5,6].

Magnetic resonance spectroscopy (MRS) is a non-invasive technique capable of probing *in vivo *changes in regional metabolites within the brain, and could be specifically employed to monitor directly alterations in brain energy metabolites including glutamine, glutamate and lactate. Previous MRS reports have demonstrated elevated glutamine+glutamate (Glx) in the basal ganglia of metabolically stable PA patients [6], while lactate has been detected in periventricular white matter [7].

The present study utilises quantitative MRS to investigate brain metabolite alterations both during metabolic stability and acute encephalopathic episodes, confirming the basal ganglia as a particular target and demonstrating novel insights in to alterations in neurotransmitter metabolites in particular glutamine and glutamate.

## Methods

### Patients

Eight children (two female) with propionic acidaemia were studied (median age (range) at first MRS, 36 (7-190) months) (see table 1). Research Ethics Committee approval and informed consent were obtained. All were diagnosed in the neonatal period, four requiring neonatal haemofiltration due to severe hyperammonaemia and metabolic acidosis. Two children had received elective orthotopic liver transplantation during early childhood. The remaining six children all received regular oral carnitine and sodium benzoate, and three received sodium phenylbutyrate, in addition to dietary protein control under management of a specialist dietetic team.

**Table 1:** Clinical features of propionic acidaemia cohort and MR Imaging findings

	Neonatal CVVH?	Liver Transplant (age, years)	Other diagnoses	Age at MRS (years)	Status at time of MRS	Metabolic Drugs & Dietary Restrictions		Acute encephalopathic episodes: complications	MRI findings	
						Maintenance	Acute		Basal ganglia	Other features
1	Yes			1.5	Stable	LC, SB, PR			Normal	
2	Yes		Hypo-throidism	1	Enceph.	SP, SB, LC, T, M, PR	SP, SB, NC	Sepsis & hyper-ammonaemia,	Acutely swelling caudate, dentate	
				1.2	Stable	SB, LC, T, PR			Resolving swelling	Delayed myelination, enlarged ventricles
				3	Enceph.	SB, LC, T, PR		Cardiomyopathy, pneumonia	Acute swelling caudate, putamen, dentate	Delayed myelination, enlarged ventricles, hippocampal atrophy
3	No		Epilepsy	9.5	Enceph.	SB, LC, PR	SB LC	Rhabdomyolysis	Cystic encephalomalacia	Enlarged ventricles/cerebral volume loss
4	Yes		Hypo-thyroidism	4	Stable	SB, SP, LC, PR			Normal	
				6	Enceph.	SB, SP, LC, PR	SP, SB, LC	Pneumonia	Acute swelling caudate + putamen	Cerebral volume loss, small hippocampi
5	Yes			0.6	Stable	SB, LC, PR			Normal	Mild ventricular dilation
6	No			3	Stable	SB, SP, LC, PR			Normal	Hippocampal sclerosis
7	No	Yes (1.3)		15	Stable	Anti-rejection			Normal	
8	No	Yes (2.5)	Myoclonic epilepsy	13	Stable	Anti-rejection LC			Normal	Mild ventricular enlargement. Unilateral mesial temporal sclerosis

MRS, magnetic resonance spectroscopy; CVVH, Continuous veno-venous haemofiltration; LC, L-carnitine; SP, Sodium Phenylbutyrate; SB, Sodium Benzoate; M, metronidazole; T, thyroxine; NC, N-Carbamylglutamate; PR, dietary protein restriction; Enceph., denotes acute encephalopathic episode

Three children had one or more severe acute episodes with encephalopathy beyond the neonatal period requiring intensive care support ("severe acute episode" hereafter). During these episodes all children required ventilatory support and continuous veno-venous haemofiltration (CVVH), and only one episode was associated with significant persistent hyperammonaemia. During these episodes three children received intravenous sodium benzoate and two received sodium phenylbutyrate.

### Magnetic Resonance Spectroscopy

MRS studies were performed concurrently with clinically indicated MRI scans at 1.5 Tesla (Siemens Symphony Magnetom, NUM4 or GE Signa Excite & HDx) facilitated by general anaesthesia as needed. Five studies from three children were acquired during severe acute episodes requiring intensive care support. Six studies from six children were acquired with elective outpatient MRI studies undertaken during metabolic stability and before any severe acute episode had occurred.

MRS was acquired using point resolved spectroscopy (PRESS) technique (echo time 30 ms and 135 ms, repetition time 1500 ms, 128 repetitions) with 2 cm-sided voxels located in the left basal ganglia and right parieto-occipital white matter. Voxels were placed in standard positions guided by T1 and T2 weighted image sequences in coronal, sagittal and axial planes. Spectra were quality-assessed to ensure appropriate voxel localisation, shimming and water suppression. Raw data were processed using LCModel [8] to generate quantitative metabolite concentrations scaled to the water signal.

Comparison was made with MRS metabolite data from a cohort of children with normal appearing MRI and similar age profile who had undergone MRI/MRS for investigation of various suspected neurodegenerative and neurocognitive conditions (white matter, n = 53, median (range) age 4.7(0.5-16.7) years; basal ganglia n = 63, 4.1(0.5-16.7) years).

### Statistical Analysis

Mean spectra were generated for each cohort for basal ganglia and white matter from LCModel fitted spectra after baseline subtraction. Spectra were compared by qualitative visual inspection.

Metabolite concentrations for each region were compared between groups by Mann Whitney nonparametric U test (SPSS Statistics 17.0), with the p-value determined by the exact 2-tailed significance, with significance level set at p < 0.05.

## Results

### Magnetic Resonance Imaging

The basal ganglia appeared normal in all children (n = 6) prior to any severe acute episode beyond the neonatal period. MRI obtained during severe acute episodes demonstrated abnormal signal in the basal ganglia variably including the caudate heads, putamen and globus pallidi and in some cases there was also evidence of acute changes in the dentate nuclei and cerebral cortex. One child demonstrated evidence of previous basal ganglia infarction relating to a previous acute encephalopathic episode, with cystic degeneration of caudate, putamen and globus pallidus. Interestingly the hippocampi appeared atrophic in seven cases. Cerebral volume loss was most marked in children with preceding severe acute episodes. The two children who had liver transplantation had normal appearing basal ganglia, cortex and white matter on MRI undertaken more than 10 years post-transplant, with one showing probable temporal mesial sclerosis.

### Magnetic Resonance Spectroscopy

**Figure 1:**
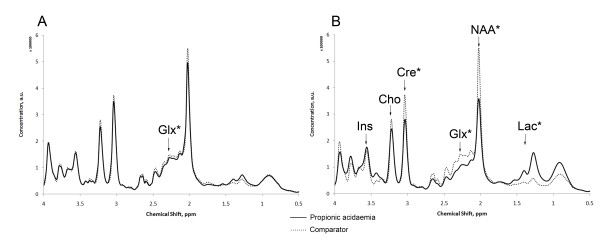
**Mean MR spectra from basal ganglia during metabolic stability and acute encephalopathy in propionic acidaemia**. (A) Metabolically stable and (B) acutely encephalopathic propionic acidaemia patients compared to normal imaging comparator cohort. ppm, parts per million; a.u., arbitrary units. Major metabolite peaks identified (labelled arrows), significant differences indicated by *. Glx, glutamine + glutamate; Ins, myo-inositol; Cre, Creatine; NAA, N-acetylaspartate; Lac, lactic acid.

Analysis of mean spectra (figure 1) and mean metabolite concentrations (table 2) demonstrate metabolite alterations in basal ganglia and parieto-occipital white matter, in particular during severe acute episodes.

**Table 2:** Metabolite concentrations in basal ganglia and parieto-occipital white matter

Basal ganglia metabolites (mM, mean (standard deviation))				
	**Propionic Acidaemia**			**Normal MRI comparator group**
	
	Metabolic stability (6 studies from 6 patients)	Acute encephalopathy (5 studies from 3 patients)	Post-liver transplant (2 studies from 2 patients)	(63 studies)
	
Median age (years, (range))	3.6 (0.6-15.9)	6.13 (1.0-9.4)	14.4 (12.9-15.9)	4.1 (0.5-16.7)
N-Acetylaspartate	5.03 (0.53)	3.89 (0.20)†#	5.35 (0.28)	5.21 (0.51)
Creatine	4.57 (0.47)	3.71 (0.58)† $	4.42 (0.14)	4.55 (0.59)
Glutamine	1.29 (0.30) *	1.00 (1.00)*	1.27 (0.25)	1.98 (0.79)
Glutamate	4.28 (0.25)	3.07 (0.60)†#	4.30 (0.39)	4.45 (0.78)
Glutamine+Glutamate	5.57 (0.41) *	4.07 (1.19)† $	5.57 (0.28)	6.43 (1.39)
myo-Inositol	2.58 (0.26)	2.79 (0.90)	2.39 (0.0)	2.49 (0.43)
Lactate	0.21 (0.25)	0.93 (0.34)†#	0.00 (0.32)	0.11 (0.20)
Choline	1.10 (0.18)	1.16 (0.29)	0.90 (0.63)	1.11 (0.18)

**White matter metabolites (mM, mean (standard deviation))**				

	**Propionic Acidaemia**			**Normal MRI comparator group**
	
	Metabolic stability (5 studies from 5 patients)	Acute encephalopathy (4 studies from 3 patients)	Post-liver transplant (2 studies from 2 patients)	(53 studies)
	
Median age (years, (range))	3.1 (0.6-15.9)	7.74 (3.0 -9.4)	14.4 (12.9-15.9)	4.7 (0.5-16.7)
N-Acetylaspartate	5.28 (0.80)	4.50 (0.47)	6.11 (0.39)	5.11 (0.73)
Creatine	3.55 (0.19) *	3.33 (0.53)	3.70 (0.00)	3.18 (0.47)
Glutamine	1.43 (0.54)	1.34 (1.30)	1.62 (0.49)	1.32 (0.62)
Glutamate	3.83 (0.45)	2.65 (0.44)*$	4.27 (0.31)	3.51 (0.78)
Glutamine+Glutamate	5.26 (0.89)	3.98 (1.54)	5.89 (0.80)	4.84 (1.18)
myo-Inositol	2.73 (0.31)	2.57 (0.39)	2.79 (0.02)	2.57 (0.39)
Lactate	0.40 (0.30)	0.81 (0.74)*	0.32 (0.45)	0.21 (0.28)
Choline	0.93 (0.15)*	1.12 (0.37)	0.83 (0.09)	1.08 (0.15)

Comparison between propionic acidaemia cohorts and normal MRI comparator group.

Mann Whitney U test (2 tailed exact significance) for difference in metabolite concentrations between Normal MRI Comparator group and propionic acidaemia cohorts,*p < 0.05, † p < 0.01. Mann Whitney U test (2 tailed exact significance) for difference in metabolite concentrations between stable and encephalopathic propionic acidaemia cohort, $ p < 0.05, # p < 0.01

MRS studies undertaken during metabolic stability before any severe acute episodes beyond the neonatal period demonstrated decreased Glx in basal ganglia compared to the normal MRI comparator group but a trend to increase in white matter. Glutamine alone was significantly decreased in basal ganglia during metabolic stability. Mean lactate was not elevated, although two cases had reliably detected lactate in basal ganglia. There was no significant decrease in the total N-acetylaspartate and N-acetylaspartylglutamate (tNAA) in this group.

MRS studies acquired during severe acute episodes demonstrated significantly decreased tNAA, Glx and creatine, and significantly elevated lactate, in the basal ganglia compared to both the normal MRI comparator group and to the metabolically stable propionic acidaemia cohort. In white matter lactate was significantly elevated but other metabolites were not significantly altered. (Data shown are for all severe acute episodes; analysis of first severe acute episode only demonstrated similar findings). There were no significant differences between groups in brain *myo-*inositol, and no propionic acid was detected in the propionic acidaemia cohort.

Separate analysis of brain metabolites from the two children post-liver transplant demonstrated normal basal ganglia tNAA and slightly decreased Glx. tNAA was elevated in white matter and total choline decreased.

## Discussion

The data presented represent the largest series of children with the severe neonatal form of propionic acidaemia to be investigated with MRS, and is to our knowledge the first to study children during severe acute encephalopathic episodes. The selective vulnerability of the basal ganglia in PA is well established, and our data provide further insight in to processes occurring within the basal ganglia. MRI results found that basal ganglia appeared normal in children who had not had a severe acute encephalopathic episode beyond the neonatal period, as similarly reported in glutaric aciduria [9], but were acutely damaged during episodes of acute illness. Similarly brain metabolite alterations were more marked during severe acute episodes.

Decreased tNAA is seen in many pathologies, and is thought to reflect loss of viable neurons [10] including following acute ischemic stroke [11]. Elevated lactate suggests a switch to anaerobic respiration, reflecting local ischaemia or mitochondrial dysfunction. As previously reported, elevated lactate was seen in two of the cases during metabolic stability, suggesting that brain tissue metabolism is not normal even when metabolism is well controlled.

The alterations seen in glutamine and glutamate in basal ganglia are of particular note. Glx was significantly decreased during severe acute episodes, with a smaller (non-significant) decrease noted in basal ganglia in studies acquired during metabolic stability. The ratio of Glx/tNAA was lower in the propionic acidaemia cohort than in cohorts of children with other defined inherited metabolic disorders including a cohort with confirmed respiratory chain disorders, while the Glx/NAA ratio was elevated in a cohort with the urea cycle disorder argininosuccinic aciduria (JED, unpublished data). This suggests that the alterations seen in Glx here were not simply a reflection of neuronal loss represented by lower tNAA.

Although the spectral signals from glutamine and glutamate overlap, there is increasing evidence that they can be differentiated in the analysis of cohorts even at 1.5Tesla using software such as LCModel. Such evidence includes Montecarlo simulations of normal brain spectra fitted with LCModel [12], comparisons of experimental and simulated basis sets [13], and the finding of a significant correlation between in vivo and in vitro results for both glutamate and glutamine detected in brain tumours [14,15].

A previous MRS study reported elevated basal ganglia Glx in three metabolically stable propionic acidaemia patients who had normal ammonia and near-normal glutamine and glutamate in plasma and cerebrospinal fluid leading the authors to suggest differential mechanisms regulating brain parenchymal metabolism [6]. However this study relied on peak area estimation and ratio to creatine, whereas the current study provides more robust quantification. Moreover the reduced glutamine detected by brain MRS is consistent with the plasma glutamine reduction previously reported in PA [16], also seen in plasma glutamine levels in our cohort (data not shown).

The administration of the alternative pathway drugs used to control hyperammonaemia depletes plasma glutamine and glycine and may contribute to the decreased brain Glx seen. Furthermore, all children were haemofiltered during severe acute episodes which may further deplete glutamine due to its high sieving coefficient [17]. However CVVH was instigated after the MRI/MRS in 80% of cases suggesting that glutamine levels were already low before CVVH was commenced.

The acute decrease seen in Glx supports the hypothesis that they are consumed to replenish a depleted Krebs cycle [4], and that this decrease is a marker of compromised aerobic cellular respiration within brain tissue. It remains to be determined whether the decreased Glx is directly detrimental or simply a secondary marker of compromised metabolic status. Interestingly the decrease in Glx was greater in the basal ganglia than in white matter, correlating with the more vulnerable brain region.

There is a need for brain protective strategies during acute metabolic decompensations, with potential methods including brain cooling, adequate energy provision, and anaplerotic therapies. MRS could provide a tool to monitor the effect of dietary supplements aimed at increasing glutamine levels, specifically monitoring metabolite levels within the target-organ of interest, namely the brain.

The two children who had liver transplant have had no severe acute encephalopathic episodes beyond the neonatal period, and have normal appearing basal ganglia on MRI. MRS demonstrated normal/high tNAA reflecting the absence of major neuronal loss and correlating with their good neurocognitive status, and no reliably detected lactate. The small numbers preclude statistical significance, but Glx was slightly increased in white matter but decreased in basal ganglia compared to the comparator group. These results support the hypothesis that liver transplantation provides systemic metabolic stability by providing a hepatic pool of functional propionyl CoA carboxylase, thus preventing further acute metabolic decompensations which are associated with the risk of brain infarction.

The data presented here have yielded some interesting observations, however further prospective data collection and analysis could overcome some of the specific limitations of the present work. In particular, use of higher field strength MRI scanners (3Tesla and above) would provide more robust differentiation of the metabolites of interest, notably glutamine and glutamate. Serial data collection in specific patients could also confirm the alterations in brain metabolites seen before, during and after metabolic decompensations. Finally analysis of a larger cohort of post-liver transplant patients as well as longer follow up with serial studies will provide stronger evidence for the therapeutic value of liver transplantation in PA.

## Conclusions

We have demonstrated that quantitative MRS is able to offer insight in to *in vivo *brain metabolic derangements, and can feasibly be employed in children with acute encephalopathy. In propionic acidaemia the use of MRS in conjunction with MRI has confirmed the basal ganglia as a particularly vulnerable target and has demonstrated novel insights in to alterations in neurotransmitter metabolites. Evaluation of patients in long-term follow up after liver transplantation demonstrates the potential for good neurological outcome and near-normal brain metabolism. MRS provides a tool to directly evaluate the tissue-level effects of novel treatment strategies including anaplerotic therapies.

## Competing interests

The authors declare that they have no competing interests.

## Authors' contributions

JED carried out data analysis and collated clinical data and drafted the manuscript.

NPD, MW, YS assisted with data processing including software development.

AC, PJM and JHW provided clinical management for subjects, assisted with patient recruitment and critically appraised the manuscript.

PG and ACP conceived of the study and participated in its design.

All authors read and approved the final manuscript.

## References

[B1] Johnson JA Le KL Palacios E Propionic acidemia: case report and review of neurologic sequelae Pediatr Neurol 2009 40 317 320 10.1016/j.pediatrneurol.2008.10.021 19302949

[B2] Barshes NR Vanatta JM Patel AJ Carter BA O'Mahony CA Karpen SJ Goss JA Evaluation and management of patients with propionic acidemia undergoing liver transplantation: a comprehensive review Pediatr Transplant 2006 10 773 781 10.1111/j.1399-3046.2006.00569.x 17032422

[B3] Schwab MA Sauer SW Okun JG Nijtmans LG Rodenburg RJ van den Heuvel LP Drose S Brandt U Hoffmann GF Ter Laak H Secondary mitochondrial dysfunction in propionic aciduria: a pathogenic role for endogenous mitochondrial toxins Biochem J 2006 398 107 112 10.1042/BJ20060221 16686602 PMC1525008

[B4] Filipowicz HR Ernst SL Ashurst CL Pasquali M Longo N Metabolic changes associated with hyperammonemia in patients with propionic acidemia Molecular Genetics and Metabolism 2006 88 123 130 10.1016/j.ymgme.2005.11.016 16406646

[B5] Ierardi-Curto L Kaplan P Saitta S Mazur A Berry GT The glutamine paradox in a neonate with propionic acidaemia and severe hyperammonaemia J Inherit Metab Dis 2000 23 85 86 10.1023/A:1005659132147 10682312

[B6] Bergman AJ Van der Knaap MS Smeitink JA Duran M Dorland L Valk J Poll-The BT Magnetic resonance imaging and spectroscopy of the brain in propionic acidemia: clinical and biochemical considerations Pediatr Res 1996 40 404 409 10.1203/00006450-199609000-00007 8865276

[B7] Chemelli AP Schocke M Sperl W Trieb T Aichner F Felber S Magnetic resonance spectroscopy (MRS) in five patients with treated propionic acidemia J Magn Reson Imaging 2000 11 596 600 10.1002/1522-2586(200006)11:6<596::AID-JMRI4>3.0.CO;2-P 10862057

[B8] Provencher SW Estimation of metabolite concentrations from localized in vivo proton NMR spectra Magn Reson Med 1993 30 672 679 10.1002/mrm.1910300604 8139448

[B9] Sauer SW Biochemistry and bioenergetics of glutaryl-CoA dehydrogenase deficiency J Inherit Metab Dis 2007 30 673 680 10.1007/s10545-007-0678-8 17879145

[B10] Griffin JL Bollard M Nicholson JK Bhakoo K Griffin JL Bollard M Nicholson JK Bhakoo K Spectral profiles of cultured neuronal and glial cells derived from HRMAS (1)H NMR spectroscopy NMR in Biomedicine 2002 15 375 384 10.1002/nbm.792 12357551

[B11] van der Zijden JP van Eijsden P de Graaf RA Dijkhuizen RM van der Zijden JP van Eijsden P de Graaf RA Dijkhuizen RM 1H/13C MR spectroscopic imaging of regionally specific metabolic alterations after experimental stroke Brain 2008 131 2209 2219 10.1093/brain/awn139 18669496

[B12] Wilson M Reynolds G Kauppinen RA Arvanitis TN Peet AC A constrained least-squares approach to the automated quantitation of in vivo (1)H magnetic resonance spectroscopy data Magn Reson Med 2011 65 1 12 10.1002/mrm.22579 20878762

[B13] Wilson M Davies NP Sun Y Natarajan K Arvanitis TN Kauppinen RA Peet AC A comparison between simulated and experimental basis sets for assessing short-TE in vivo (1)H MRS data at 1.5 T NMR Biomed 2010 23 1117 1126 10.1002/nbm.1538 20954198

[B14] Davies NP Wilson M Harris LM Natarajan K Lateef S Macpherson L Sgouros S Grundy RG Arvanitis TN Peet AC Identification and characterisation of childhood cerebellar tumours by in vivo proton MRS NMR Biomed 2008 21 908 918 10.1002/nbm.1283 18613254

[B15] Wilson M Davies NP Grundy RG Peet AC A quantitative comparison of metabolite signals as detected by in vivo MRS with ex vivo 1H HR-MAS for childhood brain tumours NMR Biomed 2009 22 213 219 10.1002/nbm.1306 19067434

[B16] Scholl-Burgi S Sass JO Heinz-Erian P Amann E Haberlandt E Albrecht U Ertl C Sigl SB Lagler F Rostasy K Karall D Changes in plasma amino acid concentrations with increasing age in patients with propionic acidemia Amino Acids 2009 10.1007/s00726-009-0356-2 19795187

[B17] Maxvold NJ Smoyer WE Custer JR Bunchman TE Amino acid loss and nitrogen balance in critically ill children with acute renal failure: a prospective comparison between classic hemofiltration and hemofiltration with dialysis Crit Care Med 2000 28 1161 1165 10.1097/00003246-200004000-00041 10809299

